# Association of neutrophil-to-lymphocyte ratio with severe radiation-induced mucositis in pharyngeal or laryngeal cancer patients: a retrospective study

**DOI:** 10.1186/s12885-021-08793-6

**Published:** 2021-09-28

**Authors:** Yumiko Kawashita, Masayasu Kitamura, Sakiko Soutome, Takashi Ukai, Masahiro Umeda, Thoshiyuki Saito

**Affiliations:** 1 Department of Oral Health, Nagasaki University Graduate School of Biomedical Sciences, Nagasaki, Japan;1-7-1 Sakamoto, Nagasaki, 852-8102 Japan; 2 Oral Management Center, Nagasaki University Hospital, Nagasaki, Japan; 3 Department of Clinical Oral Oncology, Nagasaki University Graduate School of Biomedical Sciences, Nagasaki, Japan

**Keywords:** Head and neck cancer, Radiotherapy, Mucositis, Neutrophil-to-lymphocyte ratio

## Abstract

**Background:**

The neutrophil-to-lymphocyte ratio (NLR) is a marker of systemic inflammation that informs clinical decisions regarding recurrence and overall survival in most epithelial cancers. Radiotherapy for head and neck cancer leads to mucositis in almost all patients and severe radiation-mucositis affects their quality of life (QOL). However, little is known about the NLR for severe mucositis. Therefore, this study aimed to show the association between the NLR and severe radiation-induced mucositis in hypopharyngeal or laryngeal cancer patients.

**Methods:**

In this retrospective study, we determined the incidence of grade 3 mucositis in 99 patients who were receiving definitive radiotherapy or chemoradiotherapy (CRT) for hypopharyngeal or laryngeal cancer. We performed univariate and multivariate logistic regression analyses to investigate the characteristics of grade 3 mucositis. Kaplan–Meier curves and log-rank tests were used to evaluate the occurrence of grade 3 mucositis between two groups with high (NLR > 5) or low (NLR < 5) systemic inflammation.

**Results:**

The incidence of grade 3 mucositis was 39%. Univariate logistic regression analysis showed that the NLR (Odd ratio [OR] = 1.09; 95% confidence interval [CI] = 1.02–1.16; *p* = 0.016) and smoking (OR = 1.02; 95% CI = 1.00–1.03; *p* = 0.048) were significantly associated with grade 3 mucositis. Multivariate logistic regression analysis showed that the NLR was independently associated with grade 3 mucositis (OR = 1.09; 95% CI = 1.01–1.17; *p* = 0.021). Kaplan–Meier curves also showed that patients with higher NLR (NLR > 5) prior to radiotherapy developed grade 3 mucositis more frequently than those with lower NLR during radiotherapy (*p* = 0.045).

**Conclusion:**

This study suggests that a higher NLR is a risk factor and predictor of severe radiation-induced mucositis in hypopharyngeal or laryngeal cancer patients.

## Background

Radiotherapy is a standard therapy for head and neck cancer. Radiotherapy, unlike surgery, has the advantage of organ preservation, however, mucositis usually develops as acute toxicity. Moreover, concomitant CRT induces more severe mucositis than radiotherapy alone [1, 2]. No preventive or curative treatment for radiation-induced mucositis has been established, and thus opioid-based pain control programs are generally conducted for palliative care purposes [3].

Radiation-induced oral mucositis may lead to infectious diseases: oral candidiasis [4] and aspiration pneumonia [5]. The National Comprehensive Cancer Network Guidelines® recommend oral management of radiotherapy for head and neck cancers [6]. Severe oral mucositis affects not only the QOL of patients but also the completion of radiotherapy. Opioid-based pain control programs sometimes cannot inhibit severe oral mucositis and there are opioid-related adverse events; thus, studies on topical management have been conducted. A systematic review showed that the recommendation for benzydamine mouthwash to prevent radiotherapy-induced mucositis remained unchanged [7], however, since the use of this drug is not permitted in Japan, it cannot be used.

Many cancers arise from sites of infection, chronic irritation and inflammation. The tumor microenvironment, which is largely orchestrated by inflammatory cells, is an indispensable participant in the neoplastic process, fostering proliferation, survival and migration [8]. New biomarkers of inflammatory response from serum are prognostic in many solid cancers. Elevated C-reactive protein and neutrophil-to-lymphocyte ratio (NLR) were associated with more advanced disease and poorer prognosis in oral cavity squamous cell carcinoma patients [9]. Moreover, head and neck squamous cell carcinoma cancer patients with NLR > 5 were predicted to have shorter overall survival [10].

A retrospective study showed that male sex, oropharyngeal cancer, low concentration of hemoglobin, low leukocyte or lymphocyte counts, concurrent cisplatin or cetuximab, and oral feeding were significantly associated with a higher incidence of severe oral mucositis in 326 patients with oral or oropharyngeal cancer [1]. The NLR is related to the inflammatory system, thus, high NLR may affect the severity of radiation-induced mucositis; however, this relationship has not been investigated yet. Therefore, this study was designed to describe the association between the NLR and the incidence of grade 3 mucositis.

## Methods

### Setting and design

We retrospectively reviewed the medical records of 99 patients who received oral management in association with radiotherapy for pharyngeal or laryngeal cancer at the Oral Management Center in Nagasaki University Hospital (Nagasaki, Japan) between July 2011 and June 2020. The eligibility criteria included patients who had pathologically confirmed primary mucosal squamous cell carcinoma and underwent definitive radiotherapy or CRT. Patients’ oral cavities were partially included in the irradiated area.

The trial was conducted in accordance with the Declaration of Helsinki (as revised in 2013). The study was approved by the Institutional Review Board (IRB) of Nagasaki University Hospital (approval number: 20122114). The need for informed consent was waived by the Nagasaki University Hospital Clinical Research Ethics Committee because of the retrospective nature of the study. Moreover, the research plan was published on the homepage of the participating hospitals according to the instructions of the IRB in accordance with the guaranteed opt-out opportunity.

### Outcome

The severity of radiation-induced pharyngeal or laryngeal mucositis was graded according to the National Cancer Institute Common Terminology Criteria for Adverse Events Version 5.0 (CTC v5.0) [11]. The nearest match to the grade specified in the CTC v5.0 was used. The highest grade of the severity of the mucositis during radiotherapy was recorded.

The severity of pharyngeal mucositis was as follows: grade 1, endoscopic findings only, minimal symptoms with normal oral intake, mild pain but analgesics not indicated; grade 2, moderate pain, analgesics indicated, altered oral intake, limiting instrumental activities of daily living (ADL); grade 3, severe pain, unable to adequately aliment or hydrate orally, limiting self-care ADL; grade 4, life-threatening consequences, urgent intervention indicated; and grade 5, death. The severity of laryngeal mucositis was as follows: grade 1, endoscopic findings only, mild discomfort with normal intake; grade 2, moderate pain, analgesics indicated, altered oral intake, limited instrumental automated ADL; grade 3, severe pain, severely altered eating/swallowing, medical intervention indicated; grade 4, life-threatening airway compromise, urgent intervention indicated (e.g., tracheotomy or intubation); and grade 5, death.

### Oral management associated with radiotherapy

All the patients received oral management prior to the commencement of radiotherapy [12]. Oral and dental evaluations, including panoramic X-ray examinations, were performed immediately after the introduction of doctors and infected teeth were extracted to prevent osteoradionecrosis. During radiotherapy, the teeth in dentulous patients were covered by spacers to prevent scatter radiation, particularly from metallic restorations and enamel surfaces exposed to the oral mucosa. A dental hygienist administered professional oral care at least once a week. Oral care methods included dental plaque removal via professional mechanical tooth cleaning methods and gentle removal of mucosal debris with a water-drenched sponge to keep the oral cavity as clean as possible. Oral rinsing with 4% sodium gualenate hydrate solution was prescribed at least four times daily. Oral care continued until the end of the radiotherapy treatment or hospital discharge.

### Characteristics

The following demographic and clinical characteristics were collected from the medical records: demographic factors (age, sex, body mass index [BMI], history of smoking in pack-years, Eastern Cooperative Oncology Group performance status [ECOG PS], and diabetes mellitus); tumor-related factors (primary tumor location and stage); treatment-related factors (concomitant CRT, radiotherapy method, total radiation dose, and radiation field laterality); laboratory test results (neutrophil and lymphocyte counts, and concentrations of albumin, hemoglobin, urea nitrogen and creatinine immediately before radiotherapy); and completion of radiotherapy. ECOG PS score [13] was as follows: score 0, fully active, able to carry on all pre-disease performance without restriction.; score 1, restricted in physically strenuous activity but ambulatory and able to carry out work of a light or sedentary nature, e.g., light housework, office work.; score 2, ambulatory and capable of all selfcare but unable to carry out any work activities. Up and about more than 50% of waking hours.; score 3, capable of only limited selfcare, confined to bed or chair more than 50% of waking hours; and score 4, completely disabled. Cannot carry on any selfcare. Totally confined to bed or chair. The NLR was calculated by dividing the neutrophil count by the lymphocyte count. A cut-off value of 5 was used to categorize patients with high (NLR > 5) or low (NLR < 5) systemic inflammation. This cut-off value was chosen based on a systematic review of the NLR literature on cancer, showing NLR > 5 as a predictive marker of cancer outcomes in over 30 studies of 15,500 cancer patients [14].

Concomitant chemotherapy was categorized as none (radiotherapy alone), cetuximab (bioradiotherapy [BRT]), and cisplatin or carboplatin CRT. The radiotherapy method was categorized as three-dimensional conformal radiotherapy (3D-CRT) or intensity-modulated radiotherapy (IMRT). Radiation field laterality was categorized as unilateral or bilateral. Completion of radiotherapy was categorized into three groups: completion, discontinuation, and treatment pause.

### Statistical analysis

The primary objective of this study was to determine whether NLR is associated with grade 3 mucositis. Patient characteristics were assessed for differences in radiation-induced mucositis severity (grade 1 or 2 vs. grade 3). The exploratory variables analyzed in the univariate logistic regression model were included in the multivariate logistic regression model and assessed as follows: age; BMI; pack-years of smoking; NLR; and concentrations of albumin, hemoglobin, urea nitrogen and creatinine (continuous for univariate analysis to assess linear trends); sex (male vs. female); diabetes mellitus (with vs. without); EOCG PS (0,1 vs. 2, 3); primary tumor location (hypopharynx vs. larynx); stage (I or II vs. III or IV); concomitant CRT (radiotherapy alone vs. BRT or CRT); and radiotherapy method (3D-CRT vs. IMRT). Baseline variables with *p* < 0.20 in the univariate analysis were included in the multivariable models.

The cumulative incidence of grade 3 mucositis in the two groups (NLR > 5 vs. NLR < 5) was estimated using the Kaplan–Meier method, and grade 3 mucositis estimates were compared using the log-rank test. Patients with grade 3 mucositis were calculated from the start of radiotherapy to the date of the event. Patients without grade 3 mucositis during radiotherapy were censored at the end of radiotherapy for cumulative incidence analysis.

All statistical analyses were performed using Statistical Package for the Social Sciences for Windows (version 25; IBM Corp., Tokyo, Japan). Statistical significance was defined as a two-sided *p*-value < 0.05.

## Results

### Patient demographics for the total population

A total of 99 patients were included in this retrospective study. Table 1 outlines their characteristics. Grade 3 radiation-induced mucositis occurred in 39 patients (39%). None of the patients developed grade 4 or 5 mucositis. All the patients received oral care because a part of their oral cavities was included in the radiation field.

**Table 1:** Patient characteristics

		Radiotherapy-induced mucositis			
		Grade 1&2		Grade 3	
		*N* = 60		*N* = 39	
Characteristic	Category	N	%	N	%
Sex	Male	56	93	36	92
	Female	4	7	3	8
Age	Median (25–75% tile)	65.0 (59.5–72.0)		66.0 (63.0–71.5)	
BMI (kg/m^2^)	Median (25–75% tile)	21.6 (18.7–24.0)		21.1 (19.3–24.3)	
Smoking (pack-years)	Median (25–75% tile)	29.6 (15.0–42.9)		40.0 (17.5–63.8)	
EOCG PS	0	27	45	21	54
	1	25	42	17	44
	2	6	10	1	3
	3	2	3	0	0
Diabetes mellitus	Without	47	78	32	82
	With	13	22	7	18
Primary tumor location	Hypopharynx	52	87	33	85
	Larynx	8	22	6	15
Stage	1	3	5	0	0
	2	7	12	6	15
	3	11	18	9	23
	4	39	65	24	62
Concomitant chemoradiotherapy	Radiotherapy alone	9	15	2	5
	BRT	11	18	3	8
	CRT	40	67	34	87
Total radiation dose (Gy)	Median (25–75% tile)	70.0 (63.0–70.0)		70.0 (66.0–70.0)	
Radiotherapy method	IMRT	24	40	19	49
	3D-CRT	36	60	20	51
Radiation field laterality	Bilateral	59	98	38	97
	Lateral	1	2	1	3
Completion of radiotherapy	Completion	55	92	35	90
	Discontinuation	3	5	2	5
	Treatment pause	2	3	2	5
NLR	Median (25–75% tile)	2.6 (2.0–4.7)		4.2 (2.1–11.5)	
NLR < 5		46	77	23	59
NLR > 5		14	23	16	41
albumin (g/dL)	Median (25–75% tile)	3.7 (3.5–4.2)		3.8 (3.5–4.1)	
Hemoglobin (mg/dL)	Median (25–75% tile)	13.1 (11.9–14.1)		13.3 (11.6–14.4)	
Urea nitrogen (mg/dL)	Median (25–75% tile)	13 (11–17)		14 (12–19)	
Creatinine (mg/dL)	Median (25–75% tile)	0.76 (0.67–0.92)		0.78 (0.68–0.92)	

*Abbreviations*: *BMI* Body mass index, *EOCG PS* Eastern Cooperative Oncology Group performance status, *NLR* Neutrophil-to-lymphocyte ratio, *BRT* Bioradiotherapy (cetuximab with radiotherapy), *CRT* Chemotherapy (cisplatin or carboplatin with radiotherapy), *IMRT* Intensity-modulated radiotherapy, and *3D-CRT* Three-dimensional conformal radiotherapy

### Association of NLR with grade 3 mucositis

Table 2 provides the results of the univariate analysis. The following two variables were found to be significantly associated with the occurrence of grade 3 mucositis: NLR (OR = 1.09; 95% CI = 1.02–1.16; *p* = 0.016) and smoking (OR = 1.02; 95% CI = 1.00–1.03; *p* = 0.048). Radiation field laterality was not analyzed in the logistic regression model because only two patients received radiotherapy unilaterally.

**Table 2:** Univariate analysis for grade 3 mucositis in patients receiving radiotherapy for pharyngeal or laryngeal cancer

Variable	Category (Reference)	OR	95% CI	*p*-value
Sex	Male (Female)	0.86	0.18 – 4.06	0.846
Age		1.00	0.96 – 1.05	0.894
BMI (kg/m^2^)		1.04	0.93 – 1.16	0.521
Smoking (pack-years)		1.02	1.00 – 1.03	0.048
EOCG PS	> 2 (0 or 1)	0.17	0.02 – 1.43	0.103
Diabetes mellitus	With (Without)	0.79	0.28 – 2.20	0.653
Primary tumor location	Hypopharynx (Larynx)	1.18	0.38 – 3.71	0.775
Stage	III, IV (I, II)	1.10	0.37 – 3.32	0.866
Therapy	BRT or CRT (Radiotherapy alone)	3.27	0.67 – 16.00	0.145
Radiotherapy method	IMRT (3D-CRT)	1.43	0.63 – 3.21	0.393
NLR		1.09	1.02 – 1.16	0.016
Concentration of albumin		1.42	0.62 – 3.24	0.404
Concentration of hemoglobin		1.07	0.84 – 1.35	0.593
Concentration of urea nitrogen		1.03	0.97 – 1.09	0.390
Concentration of creatinine		0.97	0.26 – 3.56	0.959

*Abbreviations*: *BMI* Body mass index, *EOCG PS* Eastern Cooperative Oncology Group performance status, *NLR* Neutrophil-to-lymphocyte ratio, *BRT* Bioradiotherapy (cetuximab with radiotherapy), *CRT* Chemotherapy (cisplatin or carboplatin with radiotherapy), *IMRT* Intensity-modulated radiotherapy, *3D-CRT* Three-dimensional conformal radiotherapy, *OR* Odds ratio, and *CI* Confidence interval

**Fig. 1:**
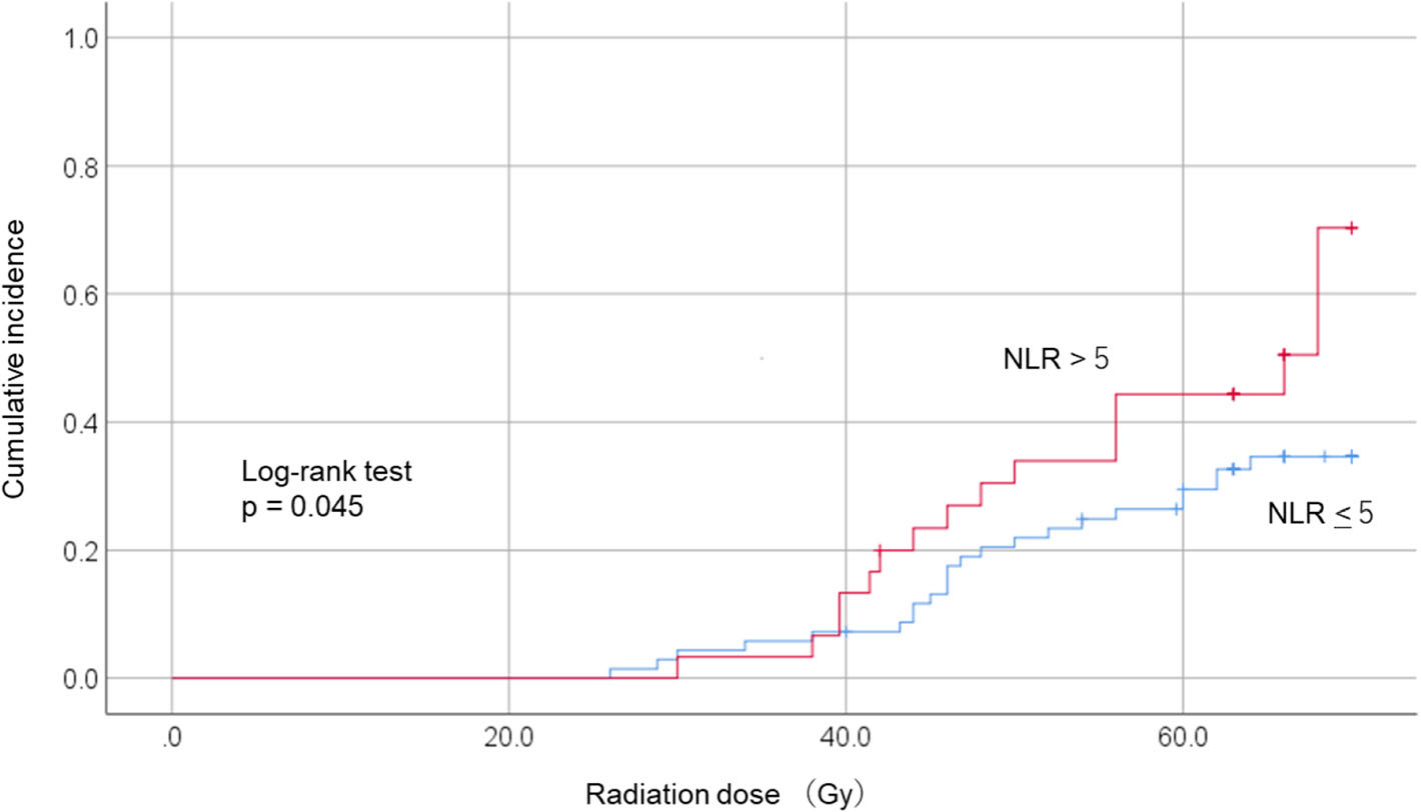
Cumulative incidence of grade 3 mucositis

Table 3 presents multivariate analysis results. NLR was significantly associated with grade 3 mucositis (OR = 1.09; 95% CI = 1.01–1.17; *p* = 0.021). Moreover, Kaplan–Meier curves showed that patients with NLR > 5 were significantly associated with the development of grade 3 mucositis (*p* = 0.045) (Fig. 1). Smoking was no longer associated with grade 3 mucositis, as adjusted by other variables.

**Table 3:** Multivariate logistic regression analysis for grade 3 mucositis in patients receiving radiotherapy for pharyngeal or laryngeal cancer

Variable	Category (Reference)	OR	95% CI	*p*-value
NLR		1.09	1.01 – 1.17	0.021
Smoking (pack-years)		1.01	1.00 – 1.03	0.133
Therapy	BRT or CRT (Radiotherapy alone)	3.29	0.58 – 19.64	0.179
EOCG PS	> 2 (0 or 1)	0.27	0.03 – 2.37	0.238

*Abbreviations*: *NLR* Neutrophil-to-lymphocyte ratio, *EOCG PS* Eastern Cooperative Oncology Group performance status, *BRT* Bioradiotherapy (cetuximab with radiotherapy), *CRT* Chemotherapy (cisplatin or carboplatin with radiotherapy), *OR* Odds ratio, and *CI* Confidence interval

## Discussion

This study investigated the outcomes for grade 3 mucositis during radiotherapy for hypopharyngeal or laryngeal cancer with adjustment for clinical characteristics. We selected hypopharyngeal or laryngeal cancer patients because radiotherapy-induced hypopharyngeal or laryngeal mucositis was not affected by teeth [12] or xerostomia [15], which were thought to have led to severe mucositis in oral or oropharyngeal cancer patients.

The results showed that the development of grade 3 mucositis was significantly associated with the NLR. Moreover, patients with a higher NLR (NLR > 5) prior to radiotherapy were significantly associated with the incidence of grade 3 mucositis during radiotherapy.

Elevated NLR was associated with decreased antitumor activity [16], tumor progression, and metastasis [8]. These results imply that tumor development may be related to the NLR imbalance in cancer patients. The NLR has been reported to be a strong predictor of mortality in patients with head and neck, esophageal, lung, breast, gastric, pancreatic, colorectal, bladder, and ovarian cancers [9, 10, 17–24]. In this study, survival analysis was not performed because the aim of our study was not survival results.

The main novel result of our study is that a higher NLR is associated with grade 3 radiation-induced mucositis. This result may be biologically plausible because neutrophils and lymphocytes are involved in the inflammatory system. In response to radiation-induced mucosal injury, a multifactorial network of chemical signals initiates and maintains a host response designed to “heal” the afflicted tissue [25]. This involves activation and directed migration of leukocytes (neutrophils, monocytes, and eosinophils), mast cells, and lymphocytes from the venous system to damage sites [8]. In head and neck squamous cell carcinoma, cancer-related inflammation is characterized by increased circulating concentrations of pro-inflammatory cytokines and C-reactive protein [9], which enhance the recruitment of circulating neutrophils, while also inhibiting the recruitment of lymphocytes for circulation [26]. These changes lead to the development of cancer-related syndromes including fever, night sweats, fatigue, cachexia, and bone and muscle pain [27]. Therefore, a higher NLR may induce severe mucositis during radiotherapy.

Evaluating NLR on the pre-radiotherapy stage may be useful to decide to conduct percutaneous endoscopic gastrostomy (PEG) before CRT. We showed that aspiration pneumonia during radiotherapy was associated with hypopharyngeal cancer, severe oral mucositis, and nasogastric tube feeding in the retrospective study. Moreover, the development of aspiration pneumonia was one of the reasons for the discontinuation of radiotherapy [5]. NCCN Guidelines Version 3.2021 [6] shows that all patients should be evaluated for nutritional risks and receive nutrition counseling by a registered dietitian and/or indicated treatment with various nutrition interventions, such as feeding tubes. However, criteria for prophylactic placement of PEG tubes before head and neck cancer treatment have not been established. If we can detect higher NLR on the pre-radiotherapy stage, we can conduct PEG before treatment, and aspiration pneumonia can be prevented during radiotherapy.

There are some limitations to this study. First, this is a single-center retrospective study with a small number of patients; therefore, it is unclear whether the results obtained can be generalized. Second, data on QOL evaluation could not be obtained because of the retrospective design of the study. However, the main strength of our study is that the study population was most likely homogeneous because the patients underwent definitive radiotherapy or CRT for hypopharyngeal or laryngeal cancer. Moreover, all patients received the same oral care management during radiotherapy because a part of their oral cavities was included in the radiation field. We would like to conduct a prospective study with a large sample size and examine QOL evaluation, such as the European Organization for Research and Treatment of Cancer QLQ-C30 or oral mucositis-specific surveys, in the future.

## Conclusions

The findings of this study suggest that a higher NLR was a risk factor for severe mucositis in patients who undergo definitive radiotherapy or CRT for hypopharyngeal or laryngeal cancer.

## Data Availability

The datasets used and/or analyzed during the current study are available from the corresponding author on reasonable request.

## Abbreviations

NLR: Neutrophil-to-lymphocyte ratio
QOL: Quality of life
CRT: Chemoradiotherapy (cisplatin or carboplatin with radiotherapy)
OR: Odds ratio
CI: Confidence interval
IRB: Institutional Review Board
CTC v5.0: National Cancer Institute Common Terminology Criteria for Adverse Events Version 5.0
ADL: Activities of daily living
BMI: Body mass index
ECOG PS: Eastern Cooperative Oncology Group performance status
BRT: Bioradiotherapy (cetuximab with radiotherapy)
IMRT: Intensity-modulated radiotherapy
3D-CRT: Three-dimensional conformal radiotherapy
